# A Case of Allergic Bronchopulmonary Aspergillosis (ABPA) in a Patient with a History of Cocaine Use and Tuberculosis

**DOI:** 10.1155/2019/3265635

**Published:** 2019-11-23

**Authors:** Noura Ayoubi, Samuel Jalali, Nikesh Kapadia

**Affiliations:** ^1^Morsani College of Medicine, University of South Florida, Tampa, FL, USA; ^2^Department of Internal Medicine, University of South Florida, Tampa, FL, USA; ^3^Department of Internal Medicine, University of South Florida, Tampa, FL, USA

## Abstract

Aspergillosis refers to a spectrum of disorders that can occur due to colonization with the *Aspergillus* fungus. Allergic bronchopulmonary aspergillosis (ABPA) is an airway hypersensitivity reaction to the fungus that is almost exclusively seen in patients with cystic fibrosis or asthma. Here, we present a case of ABPA in a patient with a history of chronic cocaine use and tuberculosis and no history of asthma or cystic fibrosis. The patient had presented with progressively worsening dyspnea for three months as well as a 20-pound weight loss. Diagnosis was made with an elevated IgE against *Aspergillus* and chest CT findings, which included bronchiectasis and tree-in-bud nodular opacities. The patient was treated with IV methylprednisolone followed by a 4-day course of oral prednisone, with significant improvement. It is our hope to make healthcare providers aware of the potential presence of ABPA in chronic cocaine users and patients with tuberculosis, both of which are not traditionally associated with this condition.

## 1. Introduction

Aspergillosis refers to a spectrum of disorders that can occur due to colonization with the *Aspergillus* fungus. Spores are commonly airborne in the environment but only classically cause disease in immunocompromised patients. *Aspergillus* is unique in the fact that it can result in either infectious or atopic manifestations. Types of aspergillosis diseases include allergic Aspergillus sinusitis, aspergilloma, invasive aspergillosis, fungal asthma, and allergic bronchopulmonary aspergillosis (ABPA) [1].

ABPA is an airway hypersensitivity reaction to the fungus that is almost exclusively seen in patients with cystic fibrosis or asthma [2, 3]. The estimated prevalence of ABPA among patients with asthma is approximately 1–2% [4]. The prevalence is slightly higher in patients with cystic fibrosis, estimating about 2–9% [5]. In rare cases, ABPA can be seen in lung transplant recipients or those with bronchiectasis, chronic granulomatous disease, or hyper-IgE syndrome [6].

The International Society for Human and Animal Mycology (ISHAM) provides a proposed set of diagnostic criteria for ABPA (Table 1) [7].These criteria are divided into three groupings: predisposing conditions, obligatory criteria, and other criteria. One of the following predisposing conditions must be present: asthma or cystic fibrosis. Both of the following obligatory criteria must be present: positive *Aspergillus* skin test or IgE levels against *Aspergillus* that are detectable and an elevated serum IgE concentration. At least 2 of the following “other criteria” must be present: precipitating *Aspergillus* serum antibodies, pulmonary opacities seen on chest radiograph, or a serum eosinophil count greater than 500 cells/microliter in patients who have not been on glucocorticoids.

Despite the requirement of one predisposing condition, ISHAM notes that a diagnosis of ABPA is still possible in patients without asthma or cystic fibrosis, albeit very rare [8]. The objective of this case report is to highlight a rare case of ABPA in a patient with a history of chronic cocaine use and tuberculosis and no history of asthma or cystic fibrosis.

## 2. Case Presentation

This patient is a 57-year-old male with a past medical history of tuberculosis and a significant social history of chronic cocaine use who presented to the emergency department due to progressively worsening dyspnea over the last three months. On physical exam, the patient had diffuse wheezing. He also endorsed a 20-pound weight loss over the last three months and increased urinary retention. The patient denied fevers, chills, recent travel, hemoptysis, and cigarette smoking.

During his hospital stay, the following labs were performed: Acid-fast bacilli smear, *Mycobacterium* polymerase chain reaction, *Legionella* antigen, urine drug screen, immunoglobulin E (IgE) levels, *Aspergillus* antigen, and neutrophilic cytoplasmic antibody. Acid-fast bacilli testing was negative, as well as mycobacterium polymerase chain reaction, *Legionella* antigen, and neutrophilic cytoplasmic antibody. Urine drug screen was positive for cocaine. Eosinophil count was elevated at 670 cells/*μ*L (normal < 610 cells/*μ*L). IgE level was also elevated at 2,378 kU/L (normal < 114 kU/L). Based on findings of elevated IgE levels, IgE against *Aspergillus fumigatus* was sought out and came back positive (0.56 kU/L).

**Figure 1:**
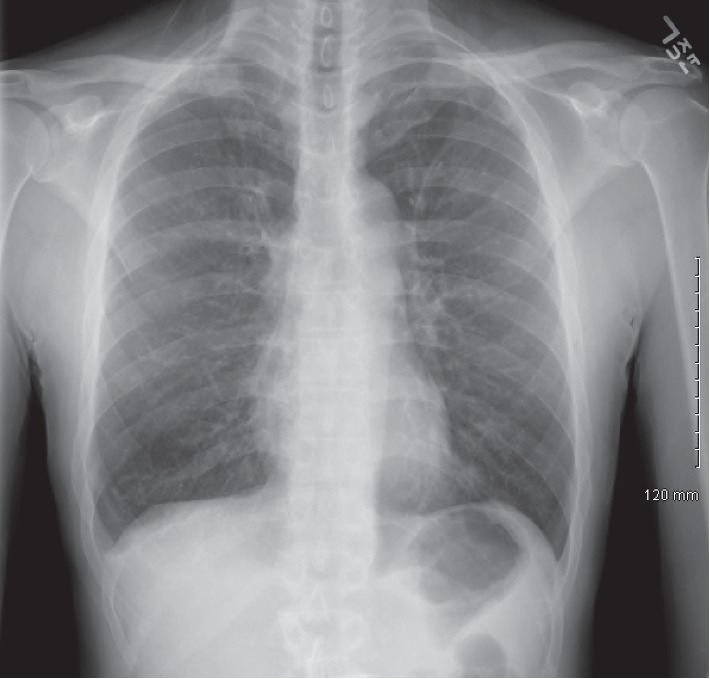
Posterior-anterior chest X-ray revealing hyperinflation, flattened diaphragm, and pleural thickening.

**Figure 2:**
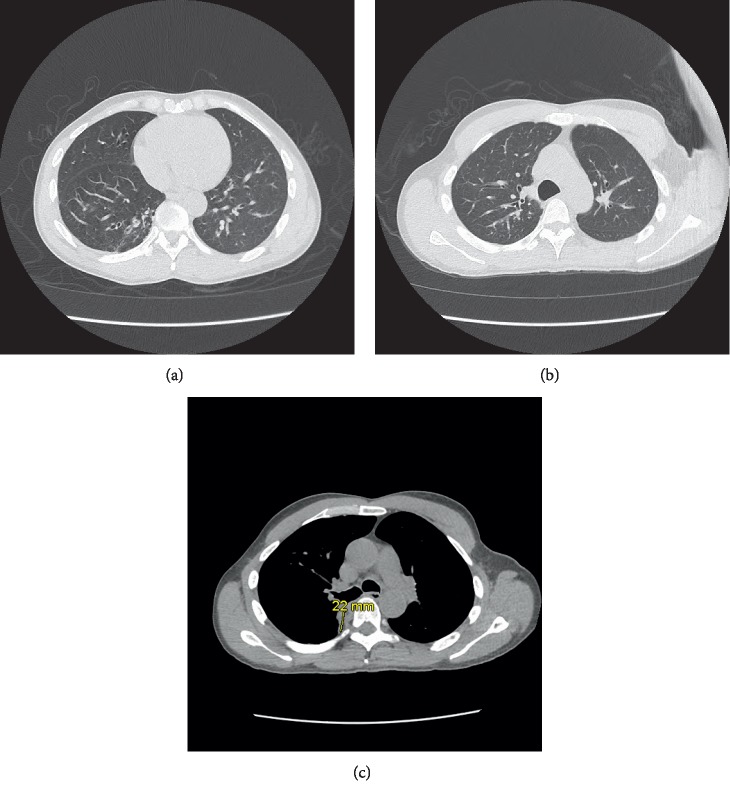
Chest CT without contrast revealing (a) bronchial wall thickening and bronchiectasis in the bilateral lower lobes; (b) bronchial wall thickening, bronchiectasis, and tree-in-bud nodularity; and (c) a 1.0 × 2.2 × 1.8 cm solid nodule in the posterior medial right lung.

Chest X-ray revealed hyperinflation, flattened diaphragm, and pleural thickening (Figure 1). Chest CT without contrast revealed some bronchial wall thickening and bronchiectasis in the bilateral lower lobes (Figure 2(a)). In the right upper lobe, there was also some bronchial wall thickening, bronchiectasis, and tree-in-bud nodularity (Figure 2(b)). A 1.0 × 2.2 × 1.8 cm solid nodule was visualized in the posterior medial right lung (Figure 2(c)). This was grossly stable in size from a chest CT 3 months prior.

The patient was first treated at an outside hospital for pneumonia with amoxicillin-clavulanate and a 7-day course of prednisone 10 mg at the first onset of his symptoms. He reported transient improvement with steroids. On presentation, prior to confirmation of ABPA, the patient was treated with 60 mg intravenous methylprednisolone BID and inhaled ipratropium bromide with albuterol sulfate. Once ABPA was confirmed, the patient was transitioned from methylprednisolone to a 4-day treatment course with 60 mg oral prednisone. The patient noted significant improvement prior to discharge.

Given the concern for malignancy due to significant weight loss and urinary retention, the prostate-specific antigen (PSA) was measured. This was elevated at 11.2 ng/ml (normal < 4.0 ng/ml). Urology was consulted, and outpatient follow-up with urology was recommended for a transrectal ultrasound- (TRUS-) guided biopsy.

## 3. Discussion

Aspergillosis refers to a spectrum of disorders that can occur due to colonization with a type of *Aspergillus* fungus, most commonly *Aspergillus fumigatus*. Spores are commonly airborne in the environment yet rarely cause disease in immunocompetent patients. On the other hand, immunocompromised patients are at risk of various presentations of disease due to inhalation of the fungus. *Aspergillus* is unique in the fact that it can result in either infectious or atopic manifestations. Types of aspergillosis infections include allergic *Aspergillus* sinusitis, aspergilloma, invasive aspergillosis, and allergic bronchopulmonary aspergillosis [1].

Allergic *Aspergillus* sinusitis (AAS) is a hypersensitivity reaction to *Aspergillus* that results in sinusitis. The reaction is similar to that of allergic bronchopulmonary aspergillosis; however, it occurs in the sinuses. Despite the similar mechanism of colonization, few cases report simultaneous presence of both diseases [9]. Patients at risk for developing AAS include those with a prior sensitization to the *Aspergillus* antigen who also have a past medical history of asthma or rhinosinusitis [10].

An aspergilloma is an intracavitary fungus ball that typically presents in previously colonized areas of the lung [11]. For this reason, many patients may have a history of colonization with *Mycobacterium tuberculosis.* [12]. Chest X-ray shows a cavitary lesion with a thickened wall. In most instances, this is an incidental finding, and patients remain asymptomatic. Rarely, patients can present with hemoptysis secondary to blood vessel invasion. *Aspergillus* sensitization has also been commonly reported in the setting of pulmonary tuberculosis cavitary disease [12]. Dhooria et al. reported that 16% of patients with a history of pulmonary tuberculosis had *Aspergillus* sensitization. Nevertheless, a diagnosis of tuberculosis is not a “predisposing condition” in the ISHAM criteria [12].

Invasive aspergillosis refers to systemic dissemination of the fungus. Normally, alveolar macrophages and epithelial cells work together to rid the lungs of infection before dissemination can occur [13]. In cases where this is inefficient, the fungi are able to latch onto blood vessels, invade the endothelium, and spread throughout the body. Risk factors for invasive aspergillosis include severe neutropenia, glucocorticoids given in high doses, AIDS, and immunosuppression [14]. Once the fungus becomes systemic, it can affect almost every organ, including the liver, kidneys, eyes, and brain [14].

Allergic bronchopulmonary aspergillosis (ABPA) is a hypersensitivity reaction to the *Aspergillus* fungus, similar to AAS. However, this reaction occurs in the airways [3]. ABPA is almost exclusively seen in patients with cystic fibrosis or asthma [2]. The estimated prevalence of ABPA among patients with asthma is approximately 1–2% [4]. The prevalence is slightly higher in patients with cystic fibrosis, estimating about 2–9% [5]. In rare cases, ABPA can be seen in lung transplant recipients or those with bronchiectasis, chronic granulomatous disease, or hyper-IgE syndrome [6].

Diagnosis of ABPA is often made using the ISHAM criteria (Table 1) [7]. This patient presented with both “obligatory criteria” (elevated serum IgE concentration and elevated IgE levels against *Aspergillus fumigatus*). He also met two of the “other criteria” (tree-in-bud nodularity and bronchiectasis and total eosinophil count > 500 cells/*μ*L). This case is unique in that the patient did not have any of the predisposing conditions. ISHAM notes that a diagnosis of ABPA can still be made despite not presenting with these conditions although this is rare.

ABPA is characterized by mucoid bronchial obstruction, resulting in wheezing and rhonchi. Given the background of hypersensitivity in this disease, patients typically have associated eosinophilia and eosinophilic pneumonia [15]. Despite the severity of *Aspergillus* colonization, the fungi do not invade bronchial walls. In most cases, the presence of fungal spores in the lungs results in the formation of IgG and IgA against the antigens. These are sufficient enough to clear the *Aspergillus* [16]. In patients with a history of atopy, additional IgE antibodies also form, manifesting as the hypersensitivity reaction of ABPA.

Similar to the pathogenesis of asthma, CD4+ helper T cells also play a role in the development of ABPA [17]. When exposed to fungus, helper T cells secrete interleukins (IL), which include IL-4, IL-5, and IL-13 [18]. In response to the interleukins, the body produces IgE and eosinophils, contributing even more so to the common finding of eosinophilia. Mucus plugs contain Charcot–Leyden crystals and eosinophils. Culture of these plugs may or may not grow the *Aspergillus* fungus [15].

Chronic airway hyperreactivity due to ABPA can result in the development of bronchiectasis. Chest radiographs often reveal thickened bronchial walls and atelectasis secondary to mucoid plugs [19]. Initial pulmonary function testing presents with an obstructive pattern of changes, including an increased residual volume (RV) and a decreased forced expiratory volume in one second (FEV1) [20]. Recommended treatment focuses on controlling inflammation and is usually a taper of systemic glucocorticoids over three to six months.

ABPA is rarely reported in patients without asthma or cystic fibrosis. This patient is unique in that he did not meet the ISHAM predisposing conditions usually required for diagnosis of ABPA. It is important to note that this is a single case of such association, which does not warrant enough evidence to claim that chronic cocaine use and previously treated tuberculosis increase risk for ABPA. Nevertheless, it is our hope to make healthcare providers aware of its potential presence in chronic cocaine users and patients with tuberculosis, both of which are not traditionally associated with this condition.

## Figures and Tables

**Table 1:** ISHAM criteria for diagnosis of ABPA.

Predisposing conditions	Requirements: 1 of 2; rarely, none are present
	Asthma
	Cystic fibrosis
Obligatory criteria	Requirements: 2 of 2
	Elevated serum IgE concentration
	Elevated IgG or IgE against *Aspergillus fumigatus*
Other criteria	Requirements: 2 of 3
	Serum eosinophil count >500 cells/microliter
	Radiographic changes consistent with ABPA
	Serum antibodies to *Aspergillus fumigatus* or elevated serum *Aspergillus* IgG

## References

[1] CDC (2019). About aspergillosis. https://www.cdc.gov/fungal/diseases/aspergillosis/definition.html.

[2] Zander D. S. (2005). Allergic bronchopulmonary aspergillosis: an overview. *Archives of Pathology & Laboratory Medicine*.

[3] Greenberger P. A. (2002). Allergic bronchopulmonary aspergillosis. *Journal of Allergy and Clinical Immunology*.

[4] Agarwal R. (2009). Allergic bronchopulmonary aspergillosis. *Chest*.

[5] Maturu V. N., Agarwal R. (2015). Prevalence of Aspergillussensitization and allergic bronchopulmonary aspergillosis in cystic fibrosis: systematic review and meta-analysis. *Clinical & Experimental Allergy*.

[6] Eppinger T. M., Greenberger P. A., White D. A., Brown A. E., Cunningham-Rundles C. (1999). Sensitization to Aspergillus species in the congenital neutrophil disorders chronic granulomatous disease and hyper-IgE syndrome. *Journal of Allergy and Clinical Immunology*.

[7] Agarwal R., Chakrabarti A., Shah A. (2013). Allergic bronchopulmonary aspergillosis: review of literature and proposal of new diagnostic and classification criteria. *Clinical & Experimental Allergy*.

[8] Glancy J. J., Elder J. L., McAleer R. (1981). Allergic bronchopulmonary fungal disease without clinical asthma. *Thorax*.

[9] Panjabi C., Shah A. (2011). AllergicAspergillussinusitis and its association with allergic bronchopulmonary aspergillosis. *Asia Pacific Allergy*.

[10] Marchiori E., Hochhegger B., Zanetti G. (2016). Intracavitary nodule. *Jornal Brasileiro de Pneumologia*.

[11] Kohno S., Kobayashi T., Kakeya H., Miyazaki Y. (2003). Pulmonary aspergilloma, diagnosis and treatment. *Kekkaku*.

[12] Dhooria S., Kumar P., Saikia B. (2014). Prevalence of Aspergillus sensitisation in pulmonary tuberculosis-related fibrocavitary disease. *The International Journal of Tuberculosis and Lung Disease*.

[13] Segal B. H. (2009). Aspergillosis. *New England Journal of Medicine*.

[14] Kauffman C. (2019). *Epidemiology and Clinical Manifestations of Invasive Aspergillosis*.

[15] Cottin V., Cordier J.-F. (2005). Eosinophilic pneumonias. *Allergy*.

[16] Greenberger P., Smith L., Hsu C., Roberts M., Liotta J. (1988). Analysis of bronchoalveolar lavage in allergic bronchopulmonary aspergillosis: divergent responses of antigen-specific antibodies and total IgE. *Journal of Allergy and Clinical Immunology*.

[17] Stevens D. A., Moss R. B., Kurup V. P. (2003). Allergic bronchopulmonary aspergillosis in cystic fibrosis-state of the art: cystic fibrosis foundation consensus conference. *Clinical Infectious Diseases*.

[18] Chauhan B., Knutsen A. P., Hutcheson P. S., Slavin R. G., Bellone C. J. (1996). T cell subsets, epitope mapping, and HLA-restriction in patients with allergic bronchopulmonary aspergillosis. *Journal of Clinical Investigation*.

[19] Buckingham S., Hansell D. (2003). Aspergillus in the lung: diverse and coincident forms. *European Radiology*.

[20] Malo J. L., Longbottom J., Mitchell J., Hawkins R., Pepys J. (1977). Studies in chronic allergic bronchopulmonary aspergillosis. 3. Immunological findings. *Thorax*.

